# Myoglobin expression by alternative transcript in different mesenchymal stem cells compartments

**DOI:** 10.1186/s13287-022-02880-6

**Published:** 2022-05-21

**Authors:** Rosella Scrima, Francesca Agriesti, Consiglia Pacelli, Claudia Piccoli, Pietro Pucci, Angela Amoresano, Olga Cela, Luigi Nappi, Tiziana Tataranni, Giorgio Mori, Pietro Formisano, Nazzareno Capitanio

**Affiliations:** 1 Department of Clinical and Experimental Medicine, University of Foggia, Foggia, Italy; 2 Laboratory of Pre-Clinical and Translational Research, IRCCS-CROB, Referral Cancer Center of Basilicata, Rionero in Vulture, PZ Italy; 3 CEINGE Advanced Biotechnology and Department of Chemical Sciences, University of Napoli Federico II, Naples, Italy; 4 Department of Medical and Surgical Sciences, University of Foggia, Foggia, Italy; 5 Department of Translational Medical Sciences, Federico II University of Naples, Naples, Italy

**Keywords:** Mesenchymal stem cells, Myoglobin, Bioenergetics, Mitochondria, Mass spectrometry, Metabolic flux analysis, Cyto-imaging

## Abstract

**Background:**

The metabolic phenotype of stem cells is increasingly recognized as a hallmark of their pluripotency with mitochondrial and oxygen-related metabolism playing a not completely defined role in this context. In a previous study, we reported the ectopic expression of myoglobin (MB) in bone marrow-derived hematopoietic stem/progenitor cells. Here, we have extended the analysis to mesenchymal stem cells (MSCs) isolated from different tissues.

**Methods:**

MSCs were isolated from human placental membrane, mammary adipose tissue and dental pulp and subjected to RT-PCR, Western blotting and mass spectrometry to investigate the expression of MB. A combination of metabolic flux analysis and cyto-imaging was used to profile the metabolic phenotype and the mitochondria dynamics in the different MSCs.

**Results:**

As for the hematopoietic stem/progenitor cells, the expression of *Mb* was largely driven by an alternative transcript with the protein occurring both in the monomer and in the dimer forms as confirmed by mass spectrometry analysis. Comparing the metabolic fluxes between neonatal placental membrane-derived and adult mammary adipose tissue-derived MSCs, we showed a significantly more active bioenergetics profile in the former that correlated with a larger co-localization of myoglobin with the mitochondrial compartment. Differences in the structure of the mitochondrial network as well as in the expression of factors controlling the organelle dynamics were also observed between neonatal and adult mesenchymal stem cells. Finally, the expression of myoglobin was found to be strongly reduced following osteogenic differentiation of dental pulp-derived MSCs, while it was upregulated following reprogramming of human fibroblasts to induce pluripotent stem cells.

**Conclusions:**

Ectopic expression of myoglobin in tissues other than muscle raises the question of understanding its function therein. Properties in addition to the canonical oxygen storage/delivery have been uncovered. Finding of Mb expressed via an alternative gene transcript in the context of different stem cells with metabolic phenotypes, its loss during differentiation and recovery in iPSCs suggest a hitherto unappreciated role of Mb in controlling the balance between aerobic metabolism and pluripotency. Understanding how Mb contributes through modulation of the mitochondrial physiology to the stem cell biology paves the way to novel perspectives in regenerative medicine as well as in cancer stem cell therapy.

**Graphical abstract:**

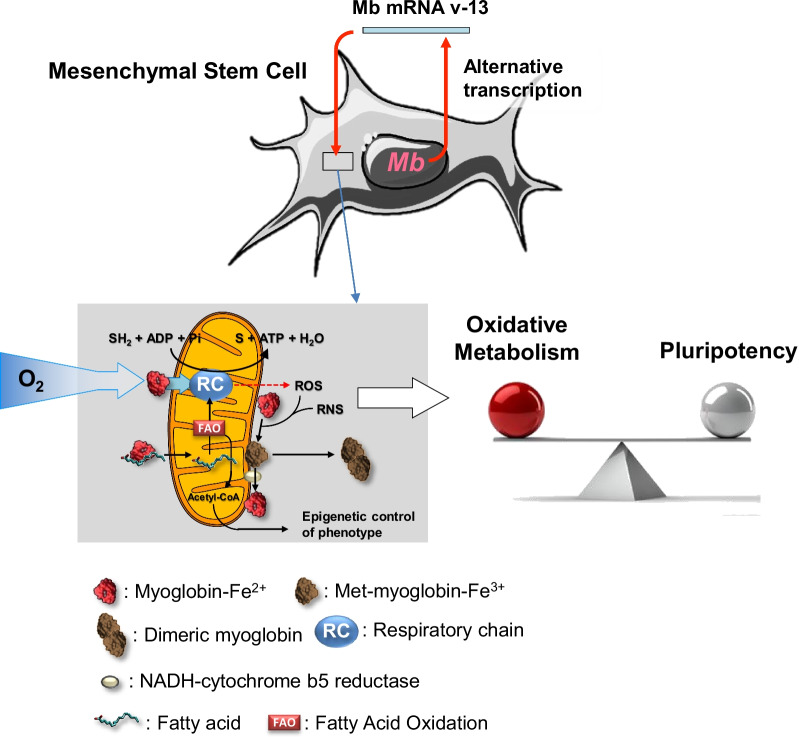

**Supplementary Information:**

The online version contains supplementary material available at 10.1186/s13287-022-02880-6.

## Background

The metabolic signature of stem cells has been receiving growing attention in the last decade because it appears not to be simply a consequence of the quiescent state of the cell. Instead, metabolic shifts proved to anticipate and influence the fate of the stem cell [1]. The metabolic phenotype of quiescent embryonic and adult stem cells is known to rely largely on glycolysis for energy production with dampening of the mitochondrial oxidative phosphorylation (OxPhos). This is thought to prevent or keep at very low level the production of reactive oxygen species (ROS), which in addition to their potential harmfulness are known to trigger spontaneous egression from the stemness state [2]. How the stem cells control their mitochondrial compartment is largely unknown. In a previous study, we found that hematopoietic progenitor/stem cells (HP/SCs) express myoglobin (Mb) which is translated from a variant of the muscular Mb mRNA [3]. HS/PCs reside in highly hypoxic environment of the bone marrow; however, given the relatively low content of Mb in HP/SCs, as compared with that of muscle cells, we questioned if its function was solely linked to di-oxygen store/deliverer. Indeed, besides its canonical function a number of additional properties of Mb have been proposed such as scavenger or generator of reactive oxygen and nitrogen species depending on the prevailing cellular/environmental conditions [4, 5]. Ectopic expression of Mb has been also found in non-muscular tissues and in a number of cancer cell lines and human tumors [6, 7].

In this study, we investigated if the expression of Mb in the context of the stemness was limited to the specific case of the HP/SCs or it was a peculiar feature of the pluripotency phenotype. To this purpose, we extended our analysis to mesenchymal stem cells sourced from different tissues.

## Methods

### Isolation and maintenance of mesenchymal stem cells

Placental amniotic membrane-derived mesenchymal stem cells (PAM-MSCs) were obtained from term placentas of healthy women after vaginal delivery or cesarean section and processed immediately. PAM-MSCs were isolated as previously described [8] and expanded by plating at a density of 10^4^/cm^2^ in AMNIOMAX medium (Thermo Fisher Scientific) [Advanced DMEM, 10% FBS, 1% Pen/Strep, 1% L-Glutamine, 1% ABAM (antibiotic–antifungal), 55 μM β-mercaptoethanol, 10 ng/ml Hr-EGF (human-recombinant epidermal growth factor)] at 37 °C in a 5% CO_2_ incubator.

Human mammary adipose tissue-derived MSCs (MAT-MSCs) were obtained from mammary adipose tissue biopsies of healthy women undergoing surgical mammary reduction. All women were otherwise healthy and free of metabolic or endocrine diseases. Adipose tissue was digested with collagenase, and MAT-MSCs were isolated as previously reported [9] and expanded in DMEM and Ham’s F12 (Thermo Fisher Scientific) (1:1) with 10% FBS, 2 mM glutamine, 100 units/ml penicillin and 100 µg/ml streptavidin.

Dental pulp MSCs (DP-MSCs) were isolated from wisdom teeth of young adult healthy volunteers as previously described [10] and expanded in mesenchymal stem cell culture medium (Thermo Fisher Scientific) supplemented with 5% heat-inactivated FBS, 100 U/mL penicillin-G, 100 µg/mL streptomycin and plated at 5 × 10^3^ cells/cm^2^.

All donors gave informed consent according to the Declaration of Helsinki, and the study was approved by the local Ethical Committees (see Declarations).

All the MSCs when the cells reached 90% of confluence were split and expanded not over the passage V.

PAM-, MAT- and DP-MSCs were characterized for the presence of mesenchymal progenitor cell surface antigens CD90, CD105 and CD73, or absence of the hematopoietic marker CD45, by cytofluorimetric analysis.

The rhabdomyosarcoma-derived cell line RD (from ATCC, CCL-136™) was used as control of Mb-expressing cell [11] and cultured in DMEM supplemented with 10% FBS.

### Flow cytometry

MSCs were suspended to a final concentration of 1.0 × 10^6^ cells/ml in phosphate-buffered saline (PBS) containing 0.1% (w/v) NaN_3_ and 5% (v/v) FBS. For specific staining, 100 μl of each MSCs suspension was incubated with 10 μl of each primary antibody for 20 min at 4 °C. The following primary antibodies were used: mouse monoclonal phycoerythrin (PE)-conjugated anti-CD73, anti-CD105, anti-CD90, anti-CD45 (BD Biosciences) and rabbit monoclonal anti-myoglobin (ab77232) from Abcam (1:50). Nonimmune, isotype-matched and isotype-conjugated mouse IgG1k and goat anti-rabbit IgG fluorescein isothiocyanate (FITC)-labeled (ab6717) antibodies were used as negative controls. For intracellular myoglobin staining, MSCs were permeabilized with Fix & Perm Kit (Life Technologies, Carlsbad, CA, USA), according to the manufacturer’s instructions, before incubation with anti-myoglobin antibody; FITC-conjugated anti-rabbit IgG was used as secondary Ab for Mb detection. Cells were assessed with Navios flow cytometer (Beckman Coulter, Indianapolis, USA); the emitted fluorescent signal of 10,000 events for each sample was acquired and analyzed using the Kaluza Analysis software (version 1.3, Beckman Coulter, Brea, CA, USA) as described [12].

### Quantitative reverse transcription polymerase chain reaction (qRT-PCR) analysis

One microgram of total RNA, isolated using Trizol reagent (Life Technologies, Paisley, UK), according to the manufacturer’s instruction and quantified on a NanoDrop spectrophotometer (Thermo Fisher Scientific, Waltham, MA, USA), was reverse-transcribed using the Transcriptor First-Strand cDNA Synthesis Kit (Roche Diagnostic, Penzberg, Germany) according to the manufacturer’s instructions. Quantitative real-time polymerase chain reaction (PCR) was performed in duplicate, using the QuantiTect Primer Assay (Qiagen, Basel, Switzerland). Quantification of the mRNA levels was performed on a LightCycler® 480 real-time PCR instrument. The relative amounts of myoglobin (variants 2 and 13), neuroglobin and cytoglobin mRNAs were normalized to GAPDH expression by LightCycler® 480 Software version 1.5 (ROCHE) using the 2^−ΔΔCt^ method. Primers and thermo-cycling parameters were as previously reported [3].

### Western blotting

Aliquots, containing 40 μg of proteins from each lysate cell, were subjected to SDS-PAGE, transferred to a PVDF membrane (Bio-Rad Laboratories, Hercules, CA, USA) using a Trans-Blot Turbo Transfer System and probed with the following primary antibodies: myoglobin, Sigma (antiserum) M8648 1:500, Abcam ab77232 1:1000, Santa Cruz sc25607 1:500; SDHB, Abcam 1:750; TOMM40, Abcam 1:1000; TOMM20, Santa Cruz 1:1000; MFN1, Santa Cruz 1:1000; MFN2, Abnova 1:1000; OPA-1, BD Bioscience 1:2000; DRP1, BD Bioscience 1:1000; LC3A/B, Cell Signaling 1:1000; Lamin, Cell Signaling 1:2000; RUNXs, Cell Signaling 1:1000; *β*-actin, Sigma 1:10,000; GAPDH, Cell Signaling 1:5000. After incubation with a correspondingly suited horseradish peroxidase-conjugated secondary antibody (1:2500; Cell Signaling Technology), signals were developed using the enhanced chemiluminescence kit (ClarityTM Western ECL Substrate, Bio-Rad), acquired by the ChemiDoc Imaging Systems XRS + (Bio-Rad) and analyzed using Image Lab software (version 4.1, Bio-Rad, Hercules, CA, USA).

### Subcellular compartment analysis

Mitochondrial, cytosolic and nuclear fractions were isolated from PAM- and MAT-MSC cells by using the Qproteome Mitochondrial Isolation Kit (Cat. No. 37612, Qiagen, Basel, Switzerland) according to the protocol provided in the kit handbook. The subcellular fractions were stored at -80 °C for further SDS-PAGE and Western blotting analysis.

### Immunoprecipitation and mass spectrometry

Immunoprecipitations of anti-myoglobin-proteins from PAM-, MAT- and DP-MSCs were carried out using Protein A Agarose (Thermo Fisher Scientific, Waltham, Massachusetts, USA) on 1 mg of total cell extracts. Lysates were pre‐cleared by incubating with protein A/G‐Agarose (Thermo Fischer cat.no 26146) for 1 h at 4 °C and then incubated with the anti-myoglobin antibody (Sigma M8648) under stirring for 18 h at 4 °C. Subsequently, samples were further incubated for 1 h at 4 °C with fresh beads. Beads were then collected by centrifugation and washed twice in lysis buffer. Samples were subjected to SDS-PAGE, and the resulting gels stained with colloidal Coomassie blue for mass spectrometry analysis. Protein bands were excised from the gel, washed twice alternatively with 50 mM ammonium bicarbonate buffer and acetonitrile and incubated with 10 μL of 10 ng/μL trypsin solution in 50 mM NH_4_HCO_3_ pH = 8 at 4 °C, for 1 h. Finally, a supplemental volume of 50 mM NH_4_HCO_3_ was added to cover the gel bands and samples were placed overnight at 37 °C.

Peptide mixtures analyses were performed by nano-LC–MS/MS by using the LTQ Orbitrap XL (Thermo Fisher) equipped with a nano-HPLC (nano-Easy II, Proxeon). After loading, each peptide mixture was first concentrated and desalted onto a pre-column (C18 EasyColumn L = 2 cm, 5 μm, ID = 100 µm, Thermo Fisher Scientific) and then fractionated on a C18 reverse-phase capillary column (C18 EasyColumn L = 10 cm, ID = 75 μm, 3 μm, Thermo Fisher Scientific) working at a flow rate of 300nL/min, with a gradient of eluent B (0,2% formic acid, 95% acetonitrile LC–MS Grade) and eluent A (0,2% formic acid, 2% acetonitrile LC–MS Grade) from 5 to 60% in 70 min. Peptide analysis was performed using data-dependent acquisition (DDA) of one MS scan (mass range from 400 to 1800 m/z) followed by CID (collision-induced dissociation) fragmentation in the ion trap of the five most abundant ions in each MS scan. Raw data from nano-LC–MS/MS analyses were processed and introduced into the MASCOT software (Matrix Science Boston, USA) to search a non-redundant protein database.

### Metabolic flux analysis

The oxygen consumption rate (OCR) and extracellular acidification rate (ECAR) were measured simultaneously in PAM- and MAT-MSCs with an XFe96 extracellular flux analyzer (Seahorse Bioscience, Billerica, MA, USA) as previously described [13]. Briefly, after attained basal (resting) activities, oligomycin (1 μM), FCCP (1 μM), rotenone + antimycin A (1 μM + 1 μM) and 2-deoxyglucose (100 mM) were injected sequentially into each well to assess the coupling of the respiratory chain, the maximal and non-mitochondrial oxygen consumption as well as basal glycolysis, glycolytic capacity and glycolytic reserve. The OCR and ECAR values were normalized to protein content in each well, determined using BCA assay (Thermo Scientific, Waltham, MA, USA). To assess the contributions to the OCR of the oxidizable substrates pyruvate/glucose, long-chain FA and glutamine, a combination of specific inhibitors was used following the protocol of the XF Mito Fuel Flex Test Kit (Seahorse—Agilent). The fuel capacity is defined as the cell’s ability to use a fuel pathway to meet metabolic demand when other fuel pathways are inhibited.

### Respirometric measurements

Cultured cells were gently detached from the dish by trypsinization, washed in PBS, harvested by centrifugation at 500 × *g* for 5 min and immediately assessed for O_2_ consumption with a high-resolution oximeter (Oxygraph-2k, Oroboros Instruments) in DMEM at 37 °C. For measurements at low oxygen concentration, the DMEM in the oxygraphic chamber was fluxed under continuous stirring with humified N_2_ to reduce the dissolved O_2_. Once the O_2_ concentration was almost zeroed, the stirring was stopped, the N_2_ inlet removed, 1–2 × 10^6^ viable cells/ml were added, the chamber stopper inserted, and the stirring re-activated. Measurement of routine OCR under this condition occurred at an O_2_ concentration of about 20–25 µM. Control assays following the same protocol but without cells ruled out the occurrence of oxygen leak in the oxygraphic chamber. The rates of oxygen consumption were corrected for 2 μM antimycin A plus 2 μM rotenone-insensitive respiration and normalized to the initial cell number or mg protein.

### Laser scanning confocal microscopy analysis

For live cell imaging of mitochondrial membrane potential (mtΔΨ), cells were cultured at low density on fibronectin-coated 35-mm glass-bottom dishes (Eppendorf, Hamburg, Germany) and incubated for 20 min at 37 °C with 2 μM of TMRE (Molecular Probes, Eugene, OR, USA). Stained cells were washed with PBS and examined using a Leica TCS SP8 confocal laser scanning microscope. For immune-cytochemistry, cells (cultured as above) were fixed, permeabilized, blocked and incubated with 1:50 diluted mouse mAb anti‐human myoglobin (Sigma) overnight at 4 °C. After two washes in PBS, the samples were incubated with 8 μg/ml of rhodamine‐labeled goat anti‐rabbit IgG (Santa Cruz). Stained cells were washed with PBS and examined using the same confocal microscope (60 × objective, 1.4 NA). Acquisition, storage and data analysis were performed with an instrumental software from Leica (LAS‐X, Wetzlar, Germany). Digitalized pictures were further processed by ImageJ (https://imagej.nih.gov)/iJ/).

### Osteogenic differentiation of dental pulp stem cells (DP-MSCs)

DP-MSCs were cultured in αMEM supplemented with 5% fetal bovine serum (FBS) and 1% penicillin–streptomycin all from GIBCO, Thermo Fisher Scientific, and incubated in 5% CO_2_ at 37 °C. Cells were harvested and subcultured according to experimental requirements. To induce osteogenic differentiation, DP-MSCs were seeded at a density of 0.5–3 × 10^5^/10-cm dish and incubated in a differentiation medium containing 10 mM dexamethasone, 10 mM *β*-glycerolphosphate, 50 µg/ml ascorbate phosphate and 2% FBS for 3 weeks. The medium was replaced every 3–4 days. The culture was stopped at different times (0–7–14–21 days), and DPSCs were harvested for analysis of myoglobin and RUNXs (osteogenic marker) expression by western blotting.

### Induced pluripotent stem cells (iPSC)

Human iPSCs were obtained as previously described [14]. Briefly, 1 × 10^5^ skin fibroblasts were nucleofected with three episomal plasmids: pCXLE-hUL (Addgene #27080), pCXLE-hSK (Addgene #27078) and pCXLE-hOCT4-shp53 (Addgene #27077), and plated in fibroblast medium for 1 week. From day 8, the cells were cultured in NutriStem XF Medium (Biological Industries, Kibbutz Beit Haemek, Israel). iPSC colonies were manually cut and passaged for expansion. Absence of mycoplasma contamination was verified by PCR analysis using EZ-PCR kit (Biological Industries, Kibbutz Beit Haemek, Israel).

### Statistical analysis

Data are shown as mean ± SEM. Data were compared by an unpaired Student’s *t* test. Differences were considered statistically significant when the *p* value was less than 0.05. All analyses were performed using GraphPad Prism (GraphPad software, v 6.01, San Diego, CA, USA).

## Results

### Mesenchymal stem cells express a transcript variant of the *Mb* gene

Mesenchymal stem cells (MSCs) were isolated from placental amniotic membrane (PAM), mammary adipose tissue (MAT) and dental pulp (DP), cultured under condition preserving their undifferentiated state and immunophenotyped by flow cytometry for the presence of MSCs markers (Additional file 1: Fig. S1) [15].

As the properties of MSCs may differ depending on their source, we thought to compare PAM-MSCs, hallmarked by a high multipotent capacity because of their early embryonic origin, with adult MSCs isolated from mammary adipose tissue (MAT-MSCs) [16].

**Fig. 1:**
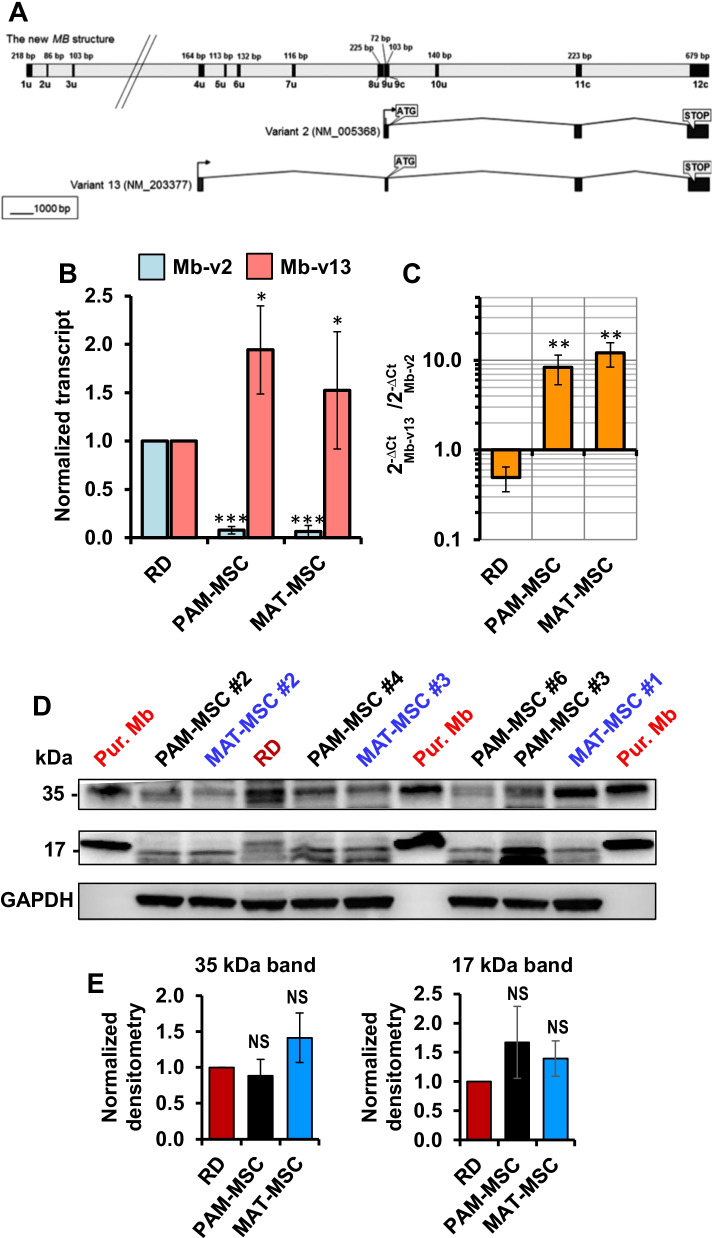
Expression of *Mb* gene transcript variants and Mb protein in PAM-MSCs and MAT-MSCs. **A** Scheme showing the structure of the alternative transcripts of the *Mb* gene; variant 2 (NM_005368) and variant 13 (NM_203377). **B** Quantitative RT-PCR analysis of the transcript variants 2 (Mb-v2) and 13 (Mb-v13) in PAM-MSCs and MAT-MSCs normalized to their expression in rhabdomyosarcoma cells (RD); means ± SEM of 4–5 independent measurements (biological replicates) each carried out in 3 technical replicates; *, *P* < 0.05 and ***, *P* < 0.001 with respect to RD. **C** Relative amounts of Mb-v13 compared to that of Mb-v2 expressed as ratios of the relative 2^−ΔCt^ values; the values shown are from the measurements in (**B**); **, *P* < 0.01 with respect to RD. **D** Comparative immunoblot for Mb detection. 40 µg protein extract from total cell lysates of four and three different preparations of PAM-MSC and MAT-MSC, respectively, was assayed by SDS-PAGE and Western blotting as described in Materials and Methods; 40 µg protein extract from rhabdomyosarcoma-derived cell line (RD) and 0.2 µg pure horse heart myoglobin (Pur. Mb) were co-analyzed for comparative purpose. **E** Densitometric analysis of comparative WBs as that shown in (D); the values refer to the 35 KDa and 17 kDa band, corresponding to the dimeric and monomeric Mb, respectively, normalized to the GAPDH band and to the value observed for the RD cells; means ± SD of six different preparations of both PAM- and MAT-MSCs; NS, not statistically significant

To verify the expression of Mb, total RNA extracts from PAM-MSCs and MAT-MSCs were subjected to q-RT-PCR amplifying two alternative transcripts of the human *Mb* gene [17]: the variant 2 (NM_005368) expressed in muscle and hearth tissue and the variant 13 (NM_203377) expressed in hematopoietic stem/progenitor cells (HSPCs) and epithelial cancer cells (Fig. 1A) [3, 6, 18]. As a comparison, we used the Mb-expressing rhabdomyosarcoma-derived cell line RD [11], which shows that both PAM-MSCs and MAT-MSCs displayed a negligible expression of the Mb transcript variant 2 as compared with the RD cells, whereas expression of variant 13 was significantly about twofold higher. Importantly, the relative expression ratio of the two Mb variants (i.e., Mb-v13/Mb-v2) was significantly higher in both PAM- and MAT-MSCs as compared to RD (Fig. 1C). To note, the expression of neuroglobin (Ngb) and cytoglobin (Cygb), two further members of the globin family [19], was at very low levels in both PAM- and MAT-MSCs (Additional file 1: Fig. S2A,B).

### Analysis of the myoglobin protein expression in mesenchymal stem cells

Western blotting analysis on both PAM-MSC and MAT-MSC lysates using commercial Abs confirmed the presence of the Mb protein (Fig. 1D and Additional file 1: Fig. S3). Notably, comparison with purified horse heart Mb showed repeatedly the occurrence of Mb prevalently in the dimeric form (with an apparent MW of ≈ 35 kDa). This observation was independent on the presence of different concentrations of the disulfide-reducing agent *β*-mercaptoethanol (data not shown). Human Mb contains only one cysteine residue in the sequence (Cys111). Densitometric analysis of both the 35 kDa and 17 kDa band did not show significant differences between the MSC samples and RD cells (Fig. 1E). At the moment, we cannot conclude if the observed SDS-resistant dimeric Mb is artifactual or reflects an in vivo alternative state of the protein. Of note, horse Mb, which lacks cysteine residues, was found to form a stable domain-swapped dimer [20].

**Fig. 2:**
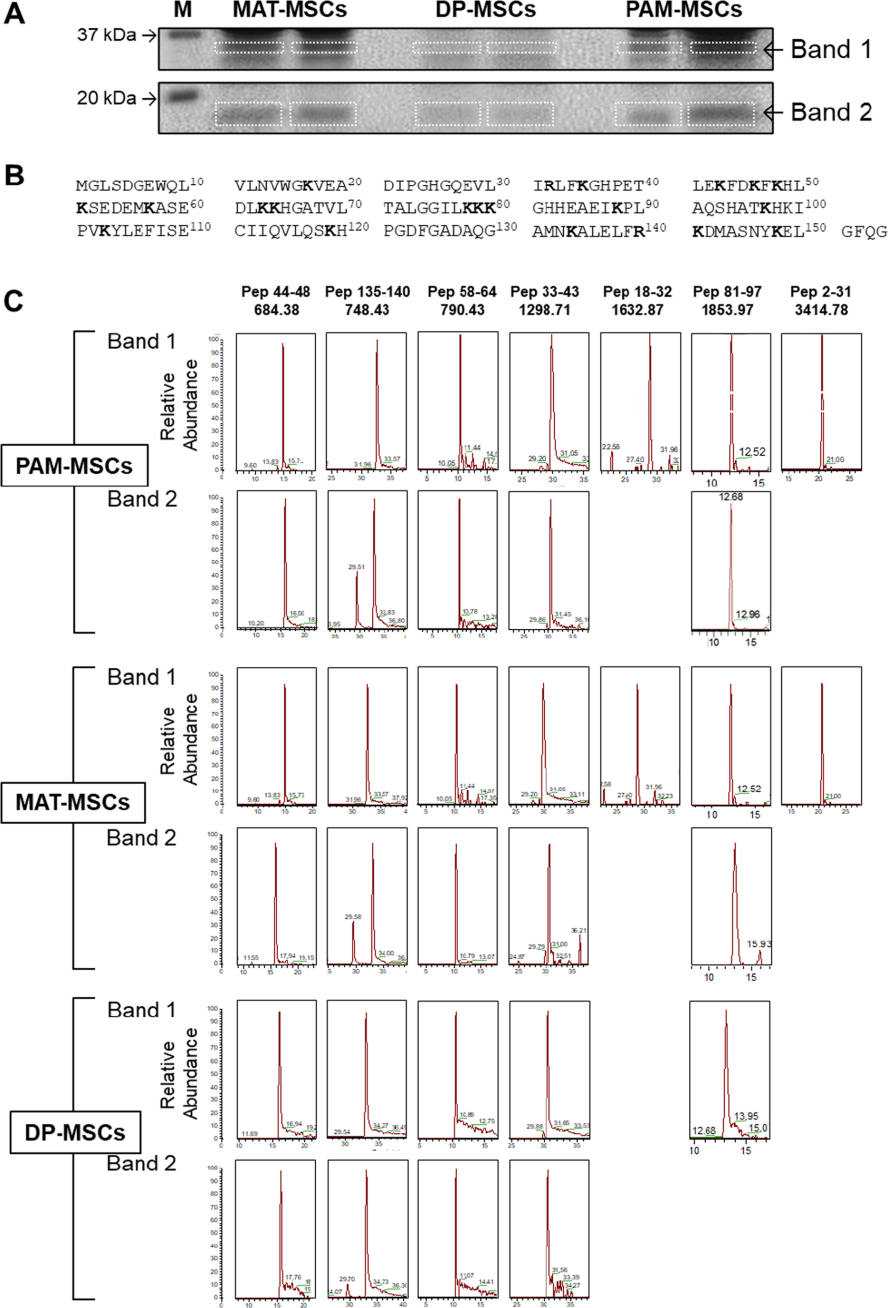
LC–MS analysis of Mb in PAM-MSCs, MAT-MSCs and DP-MSCs. **A** SDS-PAGE referring gel showing the excised bands subjected to trypsinization. **B** Sequence of human Mb with the putative sites of trypsinization indicated in bold (K, R). **C** LC–MS extract ion chromatograms identified in each of the bands indicated in (**A**) human Mb tryptic fragments; the peptide sequences and their M.W. are shown at the top of each panel lane

To provide definitive evidence of the occurrence of Mb in MSCs, we subjected the SDS-PAGE bands corresponding to the putative low and high molecular weights of Mb to trypsin digestion and mass spectrometry (MS) analysis. The results attained clearly showed, in one or the other of PAM-MSCs, MAT-MSCs and DP-MSCs, the presence of several tryptic fragments attributable to the human Mb protein sequence (Fig. 2).

The expression of Mb within the population of PAM-MSCs was evaluated assessing the co-localization of Mb and the stemness marker CD73 by flow cytometry (Additional file 1: Fig. S4A). To notice, while a large fraction of the PAM-MSCs (≈ 77%) was Mb^+^/CD73^+^ when the same analysis was carried out in MAT-MSCs, a much lower fraction of the cells (≈ 32%) resulted in Mb^+^/CD73^+^ (Additional file 1: Fig. S4B).

### Intracellular localization of myoglobin in neonatal and adult mesenchymal stem cells

**Fig. 3:**
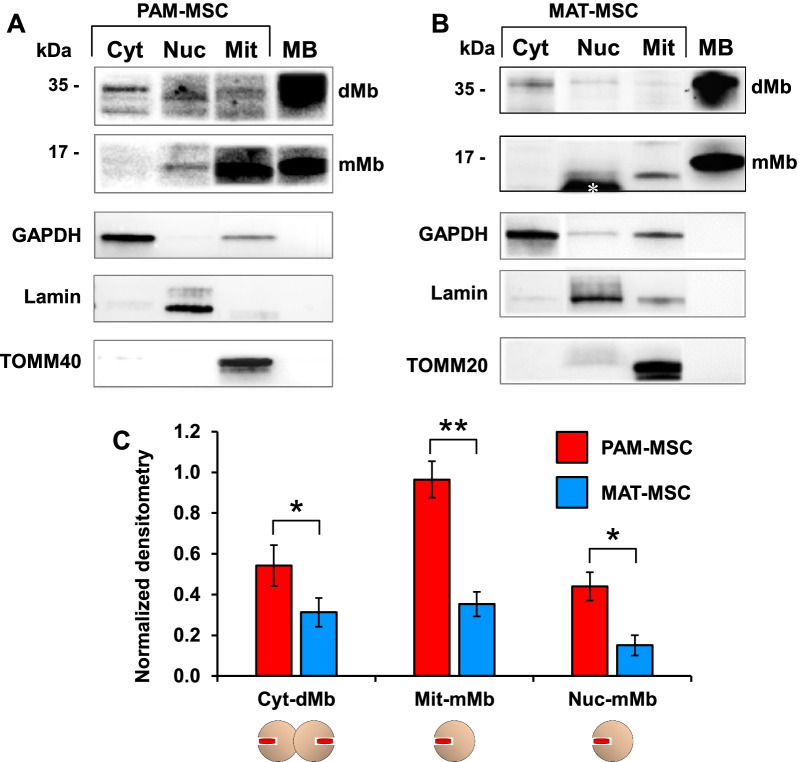
Subcellular localization of Mb. Representative immunoblots for detection of monomeric and dimeric Mb (mMb and dMB) in subcellular fractions from PAM-MSCs (**A**) and MAT-MSCs (**B**); Cyt, cytoplasmic fraction; Mit, mitochondrial fraction; Nuc, nuclear fraction. The white asterisk in (**B**) identifies a unspecific immunolabeled band. **C** Densitometric analysis; the bars are mean values ± SEM of three independent comparative experiments; GAPDH, Lamin and TOMM40 were used as internal normalizers for cytoplasmic, nuclear and mitochondrial fractions, respectively

Next, we evaluated the intracellular distribution of Mb in PAM- and MAT-MSCs. To this aim, we isolated cytoplasmic, mitochondrial and nuclear fractions from both MSCs and tested them by immunoblotting (Fig. 3). The results presented in Fig. 3A,B show that the dimeric Mb (i.e., the 35 kDa band) was largely present in the cytosolic fraction, whereas the monomeric Mb (i.e., the 17 kDa band) was present in the mitochondrial fraction and to a lesser extent in the nuclear fraction. Differences were observed between PAM-MSCs and MAT-MSCs with association of the monomeric Mb with mitochondria resulting particularly marked in PAM-MSCs (Fig. 3C).

### Mitochondrial morphology and dynamics in neonatal and adult mesenchymal stem cells

**Fig. 4:**
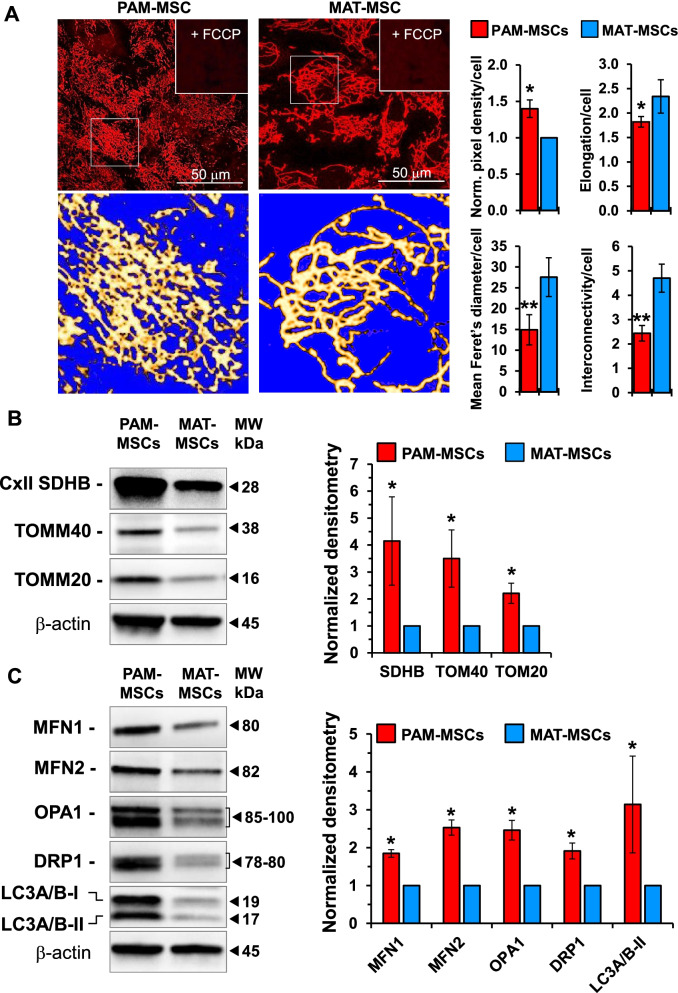
Mitochondrial morphology and dynamics in PAM-MSCs and MAT-MSCs. **A** Imaging of functional mitochondria in PAM-MSCs and MAT-MSCs**.** MSCs were incubated with TMRE and analyzed by confocal microscopy as detailed in Materials and Methods. Addition of the uncoupler FCCP vanished completely the fluorescent signal (indicated in the right upper corner of the main images). Digital enlargements of details are shown at the bottom of the pictures after false color rendering and thresholding to remove background; ImageJ tools were used with identical parameter for both PAM- and MAT-MSCs. The images shown are representative of three analyses with as many biological replicates yielding similar results. The histograms on the right show statistical morphometric analysis of the mitochondrial compartment assessed by ImageJ tools. Ten–fifteen cells/optical field from 4–5 fields of each biological replicate were randomly chosen, and the following parameters measured treating the mitochondria as objects: (1) averaged normalized TMRE pixel density/cell; (2) elongation/cell, defined as (mean perimeter)^2^/(4π × area) of all the mitochondria per cell; averaged Feret’s diameters, defined as the longest distance between any two points along the selection boundary of an object; interconnectivity/cell, defined as the (mean area)/(mean perimeter) ratio. *, *P* < 0.05; **, *P* < 0.005. **B** Representative immunoblots of mitochondrial markers. CxII SDHB, subunit B of the succinate dehydrogenase—complex II or the respiratory chain; TOMM40 and TOMM20, subunits of the translocator complex of the outer mitochondrial membrane. The histogram on the right shows the averaged normalized densitometric analysis ± S.E.M. of four independent MSCs preparations. **C** Representative immunoblots of mitochondrial dynamics markers. MFN1 and MFN2, mitofusins 1 and 2; OPA1, optic atrophy protein 1; DRP1, dynamin-related protein 1; LC3A/B-I, unmodified light chain 3A/B; LC3A/B-II, PE-conjugated light chain 3A/B. The histogram on the right shows the averaged normalized densitometric analysis ± S.E.M. of three independent MSCs preparations

On the basis of the last observed results, we sought to investigate the mitochondria-linked oxidative metabolism in the Mb differentially expressing CD73^+^ MSCs. To this aim, we first assessed the morpho-functional mitochondrial network using TMRE, a mitotropic probe which accumulates in actively respiring mitochondria driven by the mtΔψ providing, in addition, morphological indications of the organelle compartment. Figure 4A shows the comparative confocal microscopy imaging of the TMRE fluorescent signal in PAM-MSCs and MAT-MSCs; it can be clearly appreciated that while the fluorescent signal in PAM-MSCs is largely localized in a densely packed compartment, in the MAT-MSCs the fluorescence is distributed in a more rarefied filamentous interconnected network. Morphometric analysis of the images confirmed, for PAM-MSCs, a slightly higher content of the mitochondrial mass/cell but their significant lower elongation, Fetter and interconnectivity parameters as compared with MAT-MSCs. Evaluation of the expression of the succinate dehydrogenase (SDH), a marker of the respiratory chain/inner mitochondrial membrane, and of components of the translocase complex of the outer mitochondrial membranes (TOMM20 and TOMM40) would indicate a greater mitochondrial mass in PAM-MSCs than in MAT-MSCs (Fig. 4B).

It is well established that the morphology of the mitochondrial network is the result of a balance between organelle fusion and fission with its overall appearance depending on which of these processes prevails [21]. Figure 4C shows the immunoblot analysis of factors involved in the mitochondrial fusion/fission dynamics using antibodies targeting the whole protein (i.e., irrespective of its posttranslational covalent modifications). It can be appreciated that the expression of mitofusins 1 and 2 (MFN1, MFN2) and that of optic atrophy 1 protein (OPA1), involved in fusion of the outer and inner mitochondrial membranes, respectively, are both higher in PAM-MSCs as compared with MAT-MSCs. Interestingly, also the expression of the dynamin-1-related protein (DRP1), involved in mitochondrial fission, is higher in PAM-MSCs. These results would hallmark the PAM-MSCs for a more dynamic mitochondrial network. Mitochondrial dynamics is also functional to the organelle quality control; indeed, fragmentation of mitochondria facilitates the process of mitophagy which is the breakdown and recycling of damaged mitochondria [21]. Light chain 3A/B proteins (LC3A/B) are involved in the auto-/mitophagic process, and the phosphatidylethanolamine (PE)-conjugated protein (LC3A/B-II) which promotes formation of phagophores is taken as an indicator of active auto/mitophagy [22]. Consistent with this notion, the immunoblotting in Fig. 4C shows that the expression of LC3A/B-II is significantly enhanced in PAM-MSCs as compared with MAT-MSCs.

### Metabolic profile in neonatal and adult mesenchymal stem cells

**Fig. 5:**
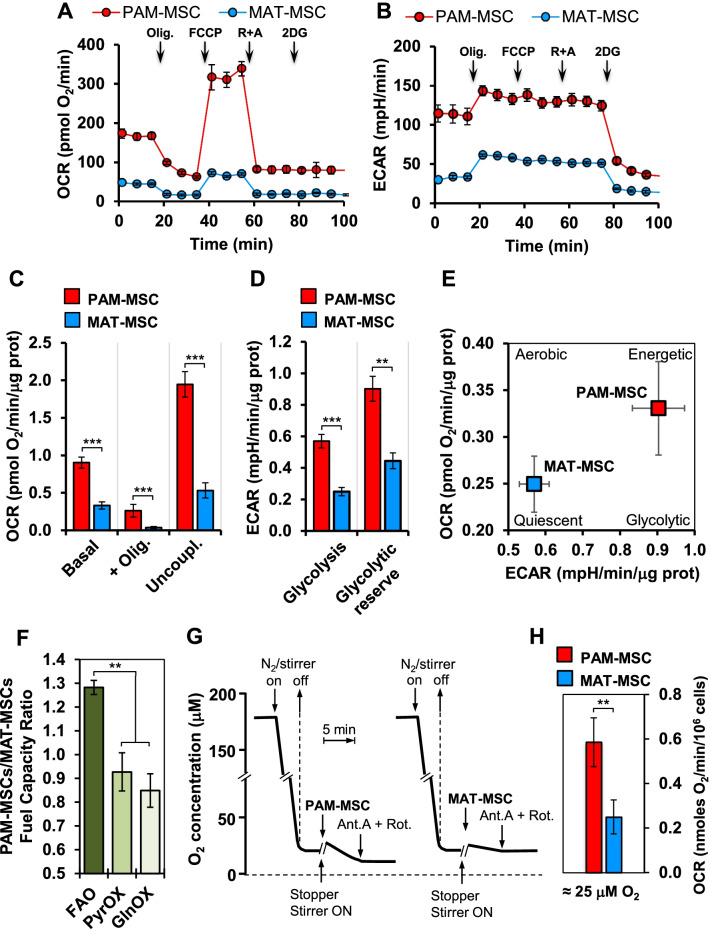
Metabolic characterization of PAM- and MAT-MSCs. Seahorse metabolic flux analyzer was used to assess the oxygen consumption rate (OCR) and the glycolysis-linked extracellular acidification rate (ECAR) in adherent PAM- and MAT-MSCs following the manufacturer protocols (see Materials and Methods). **A** and **B** show representative experimental outcomes, which indicated that oligomycin (Olig.), the uncoupler (FCCP), rotenone plus antimycin A (R + A) and 2-deoxy-glucose (2-DG) were sequentially added. **C** and **D** show the averaged values ± S.E.M. of three experiments each performed in triplicate of three independent MAT- and PAM-MSCs preparations. In (C) OCRs under basal/routine conditions (Basal), after addition of oligomycin (+ Olig.), after addition of FCCP (Uncoupl.) are shown as corrected for the R + A-insensitive OCR; ***, *P* < 0.001. In (**D**) glycolysis (i.e., basal ECAR) and glycolytic reserve (i.e., the difference between ECAR before and after addition of 2DG) are shown; ***, *P* < 0.001. **E** Bioenergetic map obtained combining basal OCR vs ECAR. **F** Relative contributions to the OCR of oxidizable substrates. The oxidative capacities of long-chain fatty acid oxidation (FAO), pyruvate oxidation (PyrOX) and glutamine oxidation (GlnOX) were assessed following the Seahorse Xf Mito Fuel Flex Test kit using combination of specific inhibitors of the three oxidative pathways as described in Materials and Methods; the capacities of each substrate were normalized to the sum of the capacities of the three substrates and the normalized capacities measured in PAM-MSCs divided by those in MAT-MSCs; the bars show the mean values ± S.E.M. of two experiments with independent MSCs preparation each performed in triplicate; **, *P* < 0.05]. **G** Representative experimental polarographic outputs of the basal O_2_ changes elicited by MAT- and PAM-MSCs after reducing the dissolved O_2_ concentration to about 20–25 µM (see Materials and Methods for details). **H** Averaged values ± S.E.M. of three experiments each performed in duplicate of three independent MAT- and PAM-MSCs preparations whose OCR was assessed as shown in panel (**G**); **, *P* < 0.01

The morphology of the mitochondrial compartment is also thought to reflect a different bioenergetic phenotype [23, 24]. Therefore, we measured, by the Seahorse methodology, the two major ATP-generating metabolic fluxes, glycolysis and mitochondrial respiration (Fig. 5A,B). The results attained clearly show that the PAM-MSCs displayed significantly higher glycolytic and respiratory fluxes, as compared with MAT-MSCs (Fig. 5C,D). Consequently, PAM-MSCs would be hallmarked by a more active bioenergetic state, whereas MAT-MSCs by a metabolic quiescent state (Fig. 5E). Interestingly, a comparison of the relative contribution to the OCR of the three major oxidizable substrates (i.e., long-chain fatty acids, glucose/pyruvate, glutamine) resulted in a significantly greater capacity ratio for fatty acid oxidation (FAO) in PAM-MSCs as compared with oxidation of the other substrates (Fig. 5F). These data are consistent with a recent report showing that FAO is an important metabolic pathway in placenta-derived MSCs [25].

To verify if the same different respiratory capacity between MAT-MSCs and PAM-MSCs was maintained under low ambient O_2_ availability, we set up a protocol to measure OCR in a Clark electrode-based oximeter at 20–25 µM O_2_ (i.e., sevenfold–ninefold lower than with the Seahorse and corresponding to about 2–3% O_2_ tension) (Fig. 5G). The results attained confirmed a significant higher respiratory competence in PAM-MSCs (Fig. 5H) also under conditions mimicking hypoxia. A plausible interpretation of these results might be linked to the different distribution of Mb in the two types of MSCs considering the function of Mb in buffering O_2_ gradients, particularly under limited O_2_ tension, that forms between the environmental peri-cellular O_2_ and the intracellular peri-mitochondrial surrounding.

To verify if chronic hypoxia was able to influence the expression of Mb, we incubated PAM-MSCs under condition of hypoxia (i.e., 0.5–1.0% O_2_) for 24 h and assessed the Mb expression by confocal immunocytochemistry imaging. Additional file 1: Figure S5A shows that following hypoxia PAM-MSCs increased a little but significantly the spotted Mb-related fluorescence/cell signal. Immunoblot analysis confirmed the moderate increased expression of Mb after 24-h incubation under hypoxia (Additional file 1: Figure S5B). To note treatment of PAM-MSCs with chetomin, an inhibitor of the HIF-1α/P300 interaction [26] did not change the expression of Mb either under normoxia or under hypoxia (Additional file 1: Figure S5B).

### Myoglobin expression following differentiation of mesenchymal stem cells and induction of pluripotent stem cells

**Fig. 6:**
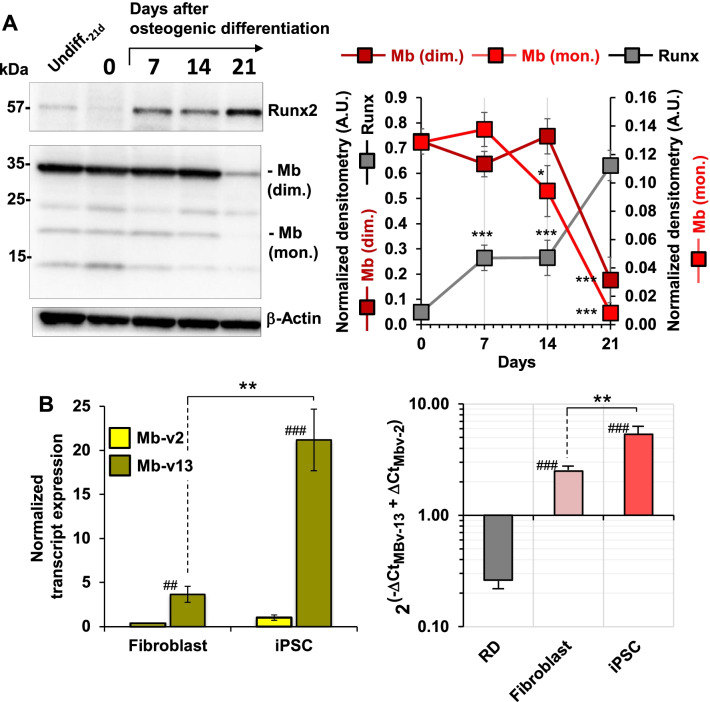
Pluripotency affects the expression of Mb. **A** Representative blotting of DP-MSCs protein extract at different times following osteogenic differentiation; bands immune-reacting for Runt-related transcription factor (RUNX2) and myoglobin (Mb) are shown; the graph on the right shows the normalized densitometric analysis of RUNX2 and of the 17 and 35 kDa Mb with values means ± S.E.M. of three independent experiments; *, *P* < 0.05, ***, *P* < 0.001 vs undifferentiated DP-MSCs. **B** Quantitative RT-PCR analysis of the transcript variants Mb-v2 and Mb-v13 in fibroblasts and fibroblast-derived iPSCs normalized to their expression in rhabdomyosarcoma cells (RD); means ± SEM of two independent measurements (biological replicates) each carried out in three technical replicates; **, *P* < 0.01 and ^##^, *P* < 0.01 and ^###^, *P* < 0.001 vs RD; the histogram on the right shows the relative amounts of Mb-v13 compared to that of Mb-v2 expressed as ratios of the relative 2^−ΔCt^ values; **, *P* < 0.01 and ^###^, *P* < 0.001 versus RD

To establish if the presence of Mb was maintained or underwent changes during differentiation of mesenchymal stem cells, we followed its expression during the osteogenic differentiation of dental pulp-derived MSCs (DP-MSCs). Figure 6A shows that the expression of the transcription factor RUNX2, a marker of osteogenic differentiation, markedly and progressively enhanced following 21 days of treatment of DP-MSCs with specific differentiation factors. Staining of the DP-MSCs with alizarin red S confirmed their advanced differentiation along the osteogenic lineage (not shown). Interestingly, the basal expression of Mb was maintained during the first 2 weeks following differentiation induction to drop markedly in the last week.

This last result suggests somehow a possible function of Mb in the maintenance of the undifferentiated state that is lost during differentiation. To test this hypothesis, as proof of principle, we decided to investigate the expression of the *Mb* gene in human fibroblast and in fibroblast-derived induced pluripotent stem cells (iPSCs) at the transcriptional level. As shown in Fig. 6B, de-differentiation of primary fibroblasts in iPSCs resulted in a significant increase in the *Mb* transcripts level in particular of the Mb-v13 variant.

## Discussion

The ectopic occurrence of Mb isoforms in tissues different from skeletal/myocardial muscle was first reported in the liver and other organs of hypoxia-tolerant fish models [27–29]. More recently, Mb was found to be expressed in several cancer cell lines and epithelial tumors [7, 30, 31] and in mice brown adipose tissue [32, 33].

In a previous report, we showed the expression of Mb in adult CD34^+^ HS/PCs [3]. In the present study, we extended our analysis to MSCs and, irrespective of their source, all cells expressed Mb at protein levels comparable with that of a muscle-derived rhabdomyosarcoma cell line (Fig. 1). The expression of Mb was conclusively proved by mass spectrometry (Fig. 2).

Alike in HS/PCs, also in MSCs, the *Mb* gene is principally transcribed by an alternative transcript (NM_203377) (Fig. 1A-C), longer than that expressed in muscle cells, likewise found in cancer cell lines as well as in different types of epithelial tumors [6, 7, 34]. The gene regulatory mechanisms driving the transcription of *Mb* in cancer cells might provide insightful in the stem cell context but remains largely elusive [18]. Several lines of evidence and bioinformatics data would suggest the presence upstream of the alternative transcription start of a putative hypoxia responsive element as well as of a hormone-receptor binding element [30]. In this study, we tested whether incubation at 0.5–1.0% oxygen of PAM-MSCs influenced Mb expression, but we found only a modest stimulatory effect (Additional file 1: Figure S5). Therefore, further studies are needed to unveil the mechanism controlling the differential transcription of the *Mb* gene in the context of stem cells.

The best-known function of Mb is to store O_2_ and regulate its bioavailability in heart and muscle to the mitochondrial compartment as the partial pressure of O_2_ drops as a consequence of increased tissue workflow or hypoxic condition. Histochemical investigations locate MSCs in perivascular niches in virtually all tissues that, however, exhibit low oxygen tension. In placental membranes, mammary glands and dental pulps around 2–3% O_2_ partial pressure (corresponding to about 15–23 mmHg) have been estimated [35, 36]. A particular case is that of hematopoietic stem cells which locate close to the endosteum of the bone marrow where 0.5% O_2_ (< 4 mmHg) is reached [36]. Therefore, it is possible that maintenance of MSCs in an undifferentiated state may require a hypoxic environment, in addition to other factors. Comparative densitometric analysis of immune blotted MSCs with pure Mb (Fig. 1D) resulted in about 10 µg Mb/mg of total protein both in PAM-MSCs and MAT-MSCs in close agreement with what found in HS/PCs [3]. This value is higher than that estimated in cancer cells [7] but at least one order of magnitude lower than that reported in human muscle cells [37]. This would suggest that in the ectopic context of MSCs the role of Mb is unrelated or not solely related to the O_2_ storage/delivery. Similar conclusion is suggested from studies where the Mb gene was overexpressed or silenced in cancer cells [38, 39].

Intriguingly, as for HS/PCs also in MSCs immunoblot analysis repeatedly resulted in appearance of a Mb dimer (about 35 kDa and confirmed by MS) in addition to the expected 17 kDa monomeric Mb (Fig. 1D and Fig. 2). The 35 kDa band was insensitive to different disulfide-reducing agents and also occurred in isolated horse heart Mb which lacks cysteine residues. The occurrence of dimeric Mb has been known for many years [40], and a later resolved X-ray crystallographic structure showed for the oxidized dimeric Mb (metMb) a domain-swapped structure with two extended α-helices [20]. Molecular simulation of the apo-myoglobin folding suggests the occurrence of additional domain-swapping arising from partially denatured proteins [41]. Aggregation of Mb was reported to occur after exposure to H_2_O_2_, causing inter-monomers cross-links between the amino acid side chains of tyrosines and tryptophans [42]. The role of scavenger for reactive oxygen (ROS) and nitrogen species (RNS) is a well-established function of Mb [43, 44], and a recent report has shown the protective role of Mb in breast cancer cells due to its ROS and NO scavenging properties [45]. The redox chemistry on which the antioxidant activity of Mb relies leads to conversion of the hematinic ferrous Mb-Fe^2+^ to the ferric species Mb-Fe^3+^. This raises the need to reduce back the ferric iron to the ferrous species in order to achieve a catalytic feature. The best characterized metMb reductase system in muscle is the NADH-cytochrome b5 oxidoreductase [46], which is localized on the ER membrane and on the outer mitochondrial membranes [47, 48]. This might account for the here-reported localization of the monomeric Mb close to mitochondria (Fig. 3 and graphical abstract).

Control of the cellular redox tone appears crucial in determining the stem cell fate [49]. Indeed, physiological levels of reactive species proved to positively contribute to adult stem cell self-renewal and pluripotency maintenance when kept at very low concentration, whereas beyond a given threshold they prompt exit of stem cells from their quiescent state, committing them to differentiation [50–52]. Several transcription factors are known to be functionally modifiable by reactive species, thereby contributing to rewiring of the stem cell transcriptome in response to redox signaling [53]. The presence of appreciable amount of monomeric Mb in the nucleus of PAM- and MAT-MSCs, here reported (Fig. 3), might serve to control the nuclear redox tone. Notably localization of Mb in the nucleus of more primitive CD34^+^-HS/PCs was shown in our previous study [3]. Cytoglobin, another member of the globin family, was also found in the cell nucleus of several mouse tissues [54] and an interactomic study of Mb unveiled its possible interaction with several nuclear proteins [55].

Analysis of the metabolic phenotype unveiled marked differences between MSCs isolated from placental membrane and mammary adipose tissue with the former exhibiting higher oxidative metabolism and glycolysis fluxes, indicating a more active metabolic state (Fig. 5A–E). This is likely to reflect the earlier embryonic origin of the PAM-MSCs, which is proved to be characterized by a significantly higher expansion capacity [56, 57]. Interestingly, comparative analysis of the contribution of the major oxidizable substrates revealed a specific larger reliance of PAM-MSCs on FAO (Fig. 5F).

Cellular metabolism is emerging as a key player for somatic stem cell behavior, determining whether a stem cell remains quiescent or starts to proliferate [58–60]. Several lines of evidence have been identified in the breakdown of lipids via FAO, a major regulator of quiescence and maintenance of the pluripotency in bone marrow hematopoietic stem cells [60], brain neural stem cells [61], skeletal muscle stem cells [62], intestinal stem cells [63, 64] and placental stem cells [25]. Reliance on FAO has also been reported in breast cancer stem cells and to be relevant for chemoresistance and recurrence [65].

The choice of fatty acid to fuel cell metabolism might be not simply linked to its greater ATP-generating efficiency. Fatty acid beta-oxidation produces significant amount of acetyl-CoA that exported outside mitochondria via the citrate shuttle can be made available for histone acetyl transferases, thereby modulating epigenetically the expression of genes involved in the stem cell identity [66–68] (see graphical abstract). Acetylation of histone H3 results in euchromatin open state which is proved to maintain the pluripotent epigenetic state and self-renewal of stem cells [69, 70]. Accordingly, it has been shown that inhibition of histone deacetylases promotes reprogramming of somatic cells in iPSCs [71].

Of relevance in the context of what above discussed is the reported evidence that Mb in its oxygenated form can bind fatty acids with affinity constants in the micromolar range and therefore compatible with physiological conditions [72–75] (see graphical abstract). Accordingly, brown adipose tissue of Mb knockout mice exhibits dysregulation of lipid homeostasis, with reduced mitochondrial density and respiratory activity [32]. All this would support the proposal that Mb might function as a transporter of fatty acid to be released to mitochondria and might account for the here-reported different levels of co-localization of Mb with mitochondria in PAM-MSCs and MAT-MSCs. Most notably, it was the monomeric form of Mb to co-localize with mitochondrial markers, whereas the dimeric Mb was found in the cytosolic fraction (Fig. 3). Interaction of Mb with the mitochondrial outer and inner membrane has been reported in muscle [76, 77] and functionally linked to the oxygen delivery of Mb to the mitochondrial respiratory chain. However, the physical interaction of Mb with mitochondria might be also linked to the aforementioned ability of Mb to vehicular fatty acids and/or to express peroxidase activity and to behave as NO dioxygenase under normoxia and as a nitrite reductase under hypoxia [5]. The mitochondrial respiratory chain is the major ROS-generating pathways in the cell [78]. At the same time, the cytochrome c oxidase of the respiratory chain is the main target of the inhibitory effect of NO on respiration [79]. On this basis, it is reasonable that the close proximity of Mb to mitochondria serves to buffer efficiently the production of reactive species right where they are generated or act. The in-cytosol-localized Mb, largely dimeric, might represent a fraction of oxidized molecules that escape re-reduction by the mitochondrial NADH-cyt.b5 reductase (see graphical abstract).

A recently emerged further function of Mb related to the mitochondrial physiology is its ability to control the mitochondrial dynamics. As reported, expression of Mb in breast cancer cells induces mitochondrial hyperfusion by upregulating mitofusins 1/2 decreasing at the same time cancer cell proliferation and tumor growth [80]. This effect would be mediated by the Mb-dependent oxidation and degradation of the E3 ubiquitin ligase parkin. The results presented in this study show that in the more primitive PAM-MSCs a denser mitochondrial mass as compared with the MAT-MSCs is accompanied with up-regulation not only of the factors involved in mitochondrial fusion but also of those involved in mitochondrial fission and in mitophagy [81] (Fig. 4). These data would suggest a more active and continuous remodeling of the mitochondrial network that would preserve the organelle quality, removing damaged mitochondria. However, at the actual stage of our knowledge we cannot conclude if the differences aforementioned between “young” and “aged” MSCs are linked to a different expression and/or mitochondrial co-localization of Mb.

Finally, it is worth highlighting that the Mb expression is lost following full differentiation of DP-MSCs along the osteogenic lineage (Fig. 6A). It remains to confirm if similar phenomena occur with different MSCs and commitments to different differentiation lineages. Along the same line, it is relevant that acquisition of stemness properties in iPSCs is accompanied with upregulation of the *Mb* gene transcript variant (Fig. 6B). All together the evidence provided in this study strongly suggests a functional role of Mb in the maintenance of pluripotency in MSCs.

## Conclusions

The main finding of our study is the definitive proof of the expression of Mb in adult stem cells by an alternative *Mb* transcript as found in several cancer cells. The Mb expression was lost during differentiation and recovered in iPSCs, suggesting its functional role in maintenance of pluripotency. Differences in the mitochondria-related metabolism and morphology between neonatal and adult MSCs might correlate with differences in the association of the monomeric Mb with mitochondria. Although we do not provide mechanistic information, the presented study suggests for Mb in MSCs a number of non-mutually exclusive possibilities encompassing fine-tuning of the cellular redox homeostasis, modulation of the mitochondrial dynamics and control of fatty acid metabolism.

## Supplementary Information

**Additional file 1**. Additional figures.

## Data Availability

All data and materials are available in the manuscript.

## Abbreviations

MB: Myoglobin
MSCs: Mesenchymal stem cells
PAM-MSCs: Placental amniotic membrane—mesenchymal stem cells
MAT-MSCs: Mammary adipose tissue—mesenchymal stem cells
DP-MSCs: Dental pulp—mesenchymal stem cells
HP/SCs: Hematopoietic progenitor/stem cells
RD: Rhabdomyosarcoma cell line
OxPhos: Oxidative phosphorylation
ROS: Reactive oxygen species
RT-PCR: Reverse transcriptase polymerase chain reaction
DMEM: Dulbecco’s modified Eagle medium
PE: Phycoerythrin
FITC: Fluorescein isothiocyanate
GAPDH: Glyceraldehyde-3-phosphate dehydrogenase
SDS-PAGE: Sodium dodecyl sulfate–polyacrylamide gel electrophoresis
SDHB: Succinate dehydrogenase complex iron sulfur subunit B
TOMM40: Translocase of outer mitochondrial membrane 40
TOMM20: Translocase of outer mitochondrial membrane 20
MFN1: Mitofusin 1
MFN2: Mitofusin 2
OPA1: Optic atrophy 1
DRP-1: Dynamin-related protein 1
LC3A/B: Light chain 3A/B
PE-LC3A/B: Phosphatidylethanolamine-conjugated LC3A/B
LC–MS/MS: Liquid chromatography tandem mass spectrometry
OCR: Oxygen consumption rate
ECAR: Extracellular acidification rate
mtΔψ: Mitochondrial membrane potential
TMRE: Tetramethylrhodamine ethyl ester
PBS: Phosphate-buffered saline
FBS: Fetal bovine serum
RUNXs: Runt-related transcription factors
iPSCs: Induced pluripotent stem cells
SEM: Standard error of the mean
Ngb: Neuroglobin
Cygb: Cytoglobin

## References

[1] Shyh-Chang N, Ng HH (2017). The metabolic programming of stem cells. Genes Dev.

[2] Piccoli C, Agriesti F, Scrima R (2013). To breathe or not to breathe: the haematopoietic stem/progenitor cells dilemma. Br J Pharmacol.

[3] D'Aprile A, Scrima R, Quarato G (2014). Hematopoietic stem/progenitor cells express myoglobin and neuroglobin: adaptation to hypoxia or prevention from oxidative stress?. Stem Cells.

[4] Flögel U, Gödecke A, Klotz LO, Schrader J (2004). Role of myoglobin in the antioxidant defense of the heart. FASEB J.

[5] Kamga C, Krishnamurthy S, Shiva S (2012). Myoglobin and mitochondria: a relationship bound by oxygen and nitric oxide. Nitric Oxide.

[6] Flonta SE, Arena S, Pisacane A, Michieli P, Bardelli A (2009). Expression and functional regulation of myoglobin in epithelial cancers. Am J Pathol.

[7] Kristiansen G, Rose M, Geisler C (2010). Endogenous myoglobin in human breast cancer is a hallmark of luminal cancer phenotype. Br J Cancer.

[8] Magatti M, Pianta S, Silini A, Parolini O (2016). Isolation, culture, and phenotypic characterization of mesenchymal stromal cells from the amniotic membrane of the human term placenta. Methods Mol Biol.

[9] D'Esposito V, Passaretti F, Hammarstedt A (2012). Adipocyte-released insulin-like growth factor-1 is regulated by glucose and fatty acids and controls breast cancer cell growth in vitro. Diabetologia.

[10] Di Benedetto A, Carbone C, Mori G (2014). Dental pulp stem cells isolation and osteogenic differentiation: a good promise for tissue engineering. Methods Mol Biol.

[11] Leader M, Patel J, Collins M, Henry K (1989). Myoglobin: an evaluation of its role as a marker of rhabdomyosarcomas. Br J Cancer.

[12] Agriesti F, Tataranni T, Pacelli C (2020). Nandrolone induces a stem cell-like phenotype in human hepatocarcinoma-derived cell line inhibiting mitochondrial respiratory activity. Sci Rep.

[13] Scrima R, Menga M, Pacelli C (2017). Para-hydroxyphenylpyruvate inhibits the pro-inflammatory stimulation of macrophage preventing LPS-mediated nitro-oxidative unbalance and immunometabolic shift. PLoS ONE.

[14] Pacelli C, Rotundo G, Lecce L (2019). Parkin mutation affects clock gene-dependent energy metabolism. Int J Mol Sci.

[15] Dominici M, Le Blanc K, Mueller I (2006). Minimal criteria for defining multipotent mesenchymal stromal cells. The International Society for Cellular Therapy position statement. Cytotherapy.

[16] Jeon YJ, Kim J, Cho JH, Chung HM, Chae JI (2016). Comparative analysis of human mesenchymal stem cells derived from bone marrow, placenta, and adipose tissue as sources of cell therapy. J Cell Biochem.

[17] Bicker A, Dietrich D, Gleixner E (2014). Extensive transcriptional complexity during hypoxia-regulated expression of the myoglobin gene in cancer. Hum Mol Genet.

[18] Bicker A, Nauth T, Gerst D (2020). The role of myoglobin in epithelial cancers: Insights from transcriptomics. Int J Mol Med.

[19] Pesce A, Bolognesi M, Bocedi A (2002). Neuroglobin and cytoglobin. Fresh blood for the vertebrate globin family. EMBO Rep.

[20] Nagao S, Osuka H, Yamada T (2012). Structural and oxygen binding properties of dimeric horse myoglobin. Dalton Trans.

[21] Westermann B (2010). Mitochondrial fusion and fission in cell life and death. Nat Rev Mol Cell Biol.

[22] Tanida I, Ueno T, Kominami E (2008). LC3 and autophagy. Methods Mol Biol.

[23] Seo BJ, Yoon SH, Do JT (2018). Mitochondrial dynamics in stem cells and differentiation. Int J Mol Sci.

[24] Chen H, Chan DC (2017). Mitochondrial dynamics in regulating the unique phenotypes of cancer and stem cells. Cell Metab.

[25] Seok J, Jung HS, Park S (2020). Alteration of fatty acid oxidation by increased CPT1A on replicative senescence of placenta-derived mesenchymal stem cells. Stem Cell Res Ther.

[26] Viziteu E, Grandmougin C, Goldschmidt H (2016). Chetomin, targeting HIF-1α/p300 complex, exhibits antitumour activity in multiple myeloma. Br J Cancer.

[27] Fraser J, de Mello LV, Ward D (2006). Hypoxia-inducible myoglobin expression in nonmuscle tissues. Proc Natl Acad Sci U S A.

[28] Cossins AR, Williams DR, Foulkes NS, Berenbrink M, Kipar A (2009). Diverse cell-specific expression of myoglobin isoforms in brain, kidney, gill and liver of the hypoxia-tolerant carp and zebrafish. J Exp Biol.

[29] Helfenrath K, Sauer M, Kamga M (2021). The more, the merrier? multiple myoglobin genes in fish species, especially in gray bichir (*Polypterus senegalus*) and reedfish (*Erpetoichthys calabaricus*). Genome Biol Evol.

[30] Bicker A, Brahmer AM, Meller S (2015). The distinct gene regulatory network of myoglobin in prostate and breast cancer. PLoS ONE.

[31] Elsherbiny ME, Shaaban M, El-Tohamy R (2021). Expression of myoglobin in normal and cancer brain tissues: correlation with hypoxia markers. Front Oncol.

[32] Aboouf MA, Armbruster J, Thiersch M (2021). Myoglobin, expressed in brown adipose tissue of mice, regulates the content and activity of mitochondria and lipid droplets. Biochim Biophys Acta Mol Cell Biol Lipids.

[33] Blackburn ML, Wankhade UD, Ono-Moore KD (2021). On the potential role of globins in brown adipose tissue: a novel conceptual model and studies in myoglobin knockout mice. Am J Physiol Endocrinol Metab.

[34] Oleksiewicz U, Daskoulidou N, Liloglou T (2011). Neuroglobin and myoglobin in non-small cell lung cancer: expression, regulation and prognosis. Lung Cancer.

[35] Mohyeldin A, Garzón-Muvdi T, Quiñones-Hinojosa A (2010). Oxygen in stem cell biology: a critical component of the stem cell niche. Cell Stem Cell.

[36] Mas-Bargues C, Sanz-Ros J, Román-Domínguez A (2019). Relevance of oxygen concentration in stem cell culture for regenerative medicine. Int J Mol Sci.

[37] Nemeth PM, Lowry OH (1984). Myoglobin levels in individual human skeletal muscle fibers of different types. J Histochem Cytochem.

[38] Galluzzo M, Pennacchietti S, Rosano S, Comoglio PM, Michieli P (2009). Prevention of hypoxia by myoglobin expression in human tumor cells promotes differentiation and inhibits metastasis. J Clin Invest.

[39] Kristiansen G, Hu J, Wichmann D (2011). Endogenous myoglobin in breast cancer is hypoxia-inducible by alternative transcription and functions to impair mitochondrial activity: a role in tumor suppression?. J Biol Chem.

[40] Van den Oord AH, Wesdorp JJ, Van Dam AF, Verheij JA (1969). Occurrence and nature of equine and bovine myoglobin dimers. Eur J Biochem.

[41] Ono K, Ito M, Hirota S, Takada S (2015). Dimer domain swapping versus monomer folding in apo-myoglobin studied by molecular simulations. Phys Chem Chem Phys.

[42] Mannino MH, Patel RS, Eccardt AM (2020). Reversible oxidative modifications in myoglobin and functional implications. Antioxidants (Basel).

[43] Richards MP (2013). Redox reactions of myoglobin. Antioxid Redox Signal.

[44] Flögel U, Merx MW, Godecke A, Decking UK, Schrader J (2001). Myoglobin: a scavenger of bioactive NO. Proc Natl Acad Sci U S A.

[45] Quinting T, Heymann AK, Bicker A (2021). Myoglobin protects breast cancer cells due to its ROS and NO scavenging properties. Front Endocrinol (Lausanne).

[46] Livingston DJ, McLachlan SJ, La Mar GN, Brown WD (1985). Myoglobin: cytochrome b5 interactions and the kinetic mechanism of metmyoglobin reductase. J Biol Chem.

[47] Borgese N, Aggujaro D, Carrera P, Pietrini G, Bassetti M (1996). A role for N-myristoylation in protein targeting: NADH-cytochrome b5 reductase requires myristic acid for association with outer mitochondrial but not ER membranes. J Cell Biol.

[48] Arihara K, Cassens RG, Greaser ML, Luchansky JB, Mozdziak PE (1995). Localization of metmyoglobin-reducing enzyme (NADH-cytochrome b(5) reductase) system components in bovine skeletal muscle. Meat Sci.

[49] Sinenko SA, Starkova TY, Kuzmin AA, Tomilin AN (2021). Physiological signaling functions of reactive oxygen species in stem cells: from flies to man. Front Cell Dev Biol.

[50] Liang R, Ghaffari S (2014). Stem cells, redox signaling, and stem cell aging. Antioxid Redox Signal.

[51] Chaudhari P, Ye Z, Jang YY (2014). Roles of reactive oxygen species in the fate of stem cells. Antioxid Redox Signal.

[52] Tan DQ, Suda T (2018). Reactive oxygen species and mitochondrial homeostasis as regulators of stem cell fate and function. Antioxid Redox Signal.

[53] Hopkins BL, Neumann CA (2019). Redoxins as gatekeepers of the transcriptional oxidative stress response. Redox Biol.

[54] Geuens E, Brouns I, Flamez D (2003). A globin in the nucleus!. J Biol Chem.

[55] Haines BA, Davis DA, Zykovich A (2012). Comparative protein interactomics of neuroglobin and myoglobin. J Neurochem.

[56] Barlow S, Brooke G, Chatterjee K (2008). Comparison of human placenta- and bone marrow-derived multipotent mesenchymal stem cells. Stem Cells Dev.

[57] Wu M, Zhang R, Zou Q (2018). Comparison of the biological characteristics of mesenchymal stem cells derived from the human placenta and umbilical cord. Sci Rep.

[58] Folmes CD, Terzic A (2016). Energy metabolism in the acquisition and maintenance of stemness. Semin Cell Dev Biol.

[59] Chandel NS, Jasper H, Ho TT, Passegué E (2016). Metabolic regulation of stem cell function in tissue homeostasis and organismal ageing. Nat Cell Biol.

[60] Ito K, Carracedo A, Weiss D (2012). A PML–PPAR-δ pathway for fatty acid oxidation regulates hematopoietic stem cell maintenance. Nat Med.

[61] Knobloch M, Pilz GA, Ghesquière B (2017). A fatty acid oxidation-dependent metabolic shift regulates adult neural stem cell activity. Cell Rep.

[62] Ryall JG, Dell'Orso S, Derfoul A (2015). The NAD(+)-dependent SIRT1 deacetylase translates a metabolic switch into regulatory epigenetics in skeletal muscle stem cells. Cell Stem Cell.

[63] Beyaz S, Mana MD, Roper J (2016). High-fat diet enhances stemness and tumorigenicity of intestinal progenitors. Nature.

[64] Mihaylova MM, Cheng CW, Cao AQ (2018). Fasting activates fatty acid oxidation to enhance intestinal stem cell function during homeostasis and aging. Cell Stem Cell.

[65] Wang T, Fahrmann JF, Lee H (2018). JAK/STAT3-regulated fatty acid β-oxidation is critical for breast cancer stem cell self-renewal and chemoresistance. Cell Metab.

[66] He R, Dantas A, Riabowol K (2021). Histone acetyltransferases and stem cell identity. Cancers (Basel).

[67] Clémot M, Sênos Demarco R, Jones DL (2020). Lipid mediated regulation of adult stem cell behavior. Front Cell Dev Biol.

[68] Zhang H, Badur MG, Divakaruni AS (2016). Distinct metabolic states can support self-renewal and lipogenesis in human pluripotent stem cells under different culture conditions. Cell Rep.

[69] Azuara V, Perry P, Sauer S (2006). Chromatin signatures of pluripotent cell lines. Nat Cell Biol.

[70] Gaspar-Maia A, Alajem A, Meshorer E, Ramalho-Santos M (2011). Open chromatin in pluripotency and reprogramming. Nat Rev Mol Cell Biol.

[71] Mali P, Chou BK, Yen J (2010). Butyrate greatly enhances derivation of human induced pluripotent stem cells by promoting epigenetic remodeling and the expression of pluripotency-associated genes. Stem Cells.

[72] Sriram R, Kreutzer U, Shih L, Jue T (2008). Interaction of fatty acid with myoglobin. FEBS Lett.

[73] Shih L, Chung Y, Sriram R, Jue T (2014). Palmitate interaction with physiological states of myoglobin. Biochim Biophys Acta.

[74] Chintapalli SV, Anishkin A, Adams SH (2018). Exploring the entry route of palmitic acid and palmitoylcarnitine into myoglobin. Arch Biochem Biophys.

[75] Hendgen-Cotta U, Esfeld D, Coman C (2017). A novel physiological role for cardiac myoglobin in lipid metabolism. Sci Rep.

[76] Postnikova GB, Shekhovtsova EA (2012). Fluorescence studies on the interaction of myoglobin with mitochondria. Biochemistry (Mosc).

[77] Yamada T, Furuichi Y, Takakura H (1985). Interaction between myoglobin and mitochondria in rat skeletal muscle. J Appl Physiol.

[78] Murphy MP (2009). How mitochondria produce reactive oxygen species. Biochem J.

[79] Brunori M (2001). Nitric oxide moves myoglobin centre stage. Trends Biochem Sci.

[80] Braganza A, Quesnelle K, Bickta J (2019). Myoglobin induces mitochondrial fusion, thereby inhibiting breast cancer cell proliferation. J Biol Chem.

[81] Ni HM, Williams JA, Ding WX (2015). Mitochondrial dynamics and mitochondrial quality control. Redox Biol.

