# Supplementary material for: Myoglobin expression by alternative transcript in different mesenchymal stem cells compartments
Source: Stem Cell Res Ther. 2022 May 21;13:209. doi: 10.1186/s13287-022-02880-6 (PMC9123686; doi:10.1186/s13287-022-02880-6)
Supplement: Supplementary file 1 — Additional file 1. Additional figures. [file 13287_2022_2880_MOESM1_ESM.docx]

**SUPPLEMENTAL DATA**

**
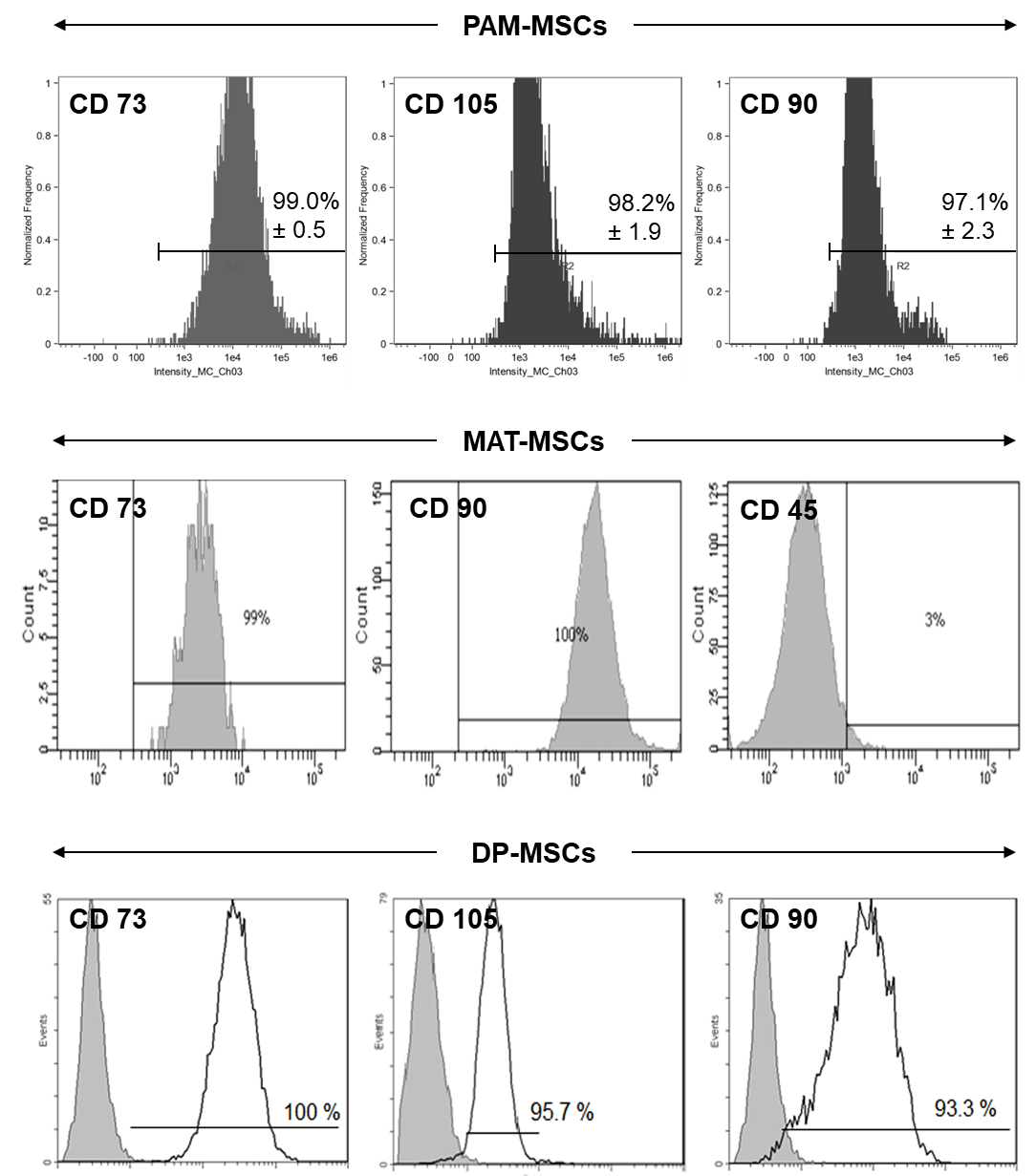
**

**Figure S1.** Immunophenotype of human mesenchymal stem cells isolated from amniotic placental membrane (PAM-MSCs), mammary adipose tissue (MAT-MSCs) and dental pulp (DP-MSCs). Cells were stained with antibodies against the indicated surface specific markers (green area) or an isotype-matched monoclonal antibody and analyzed by flow cytometry as detailed in Materials and Methods. The shown flow cytometry surface markers plots are representatives of at least four independent MSCs preparations from each of the indicated tissue source.


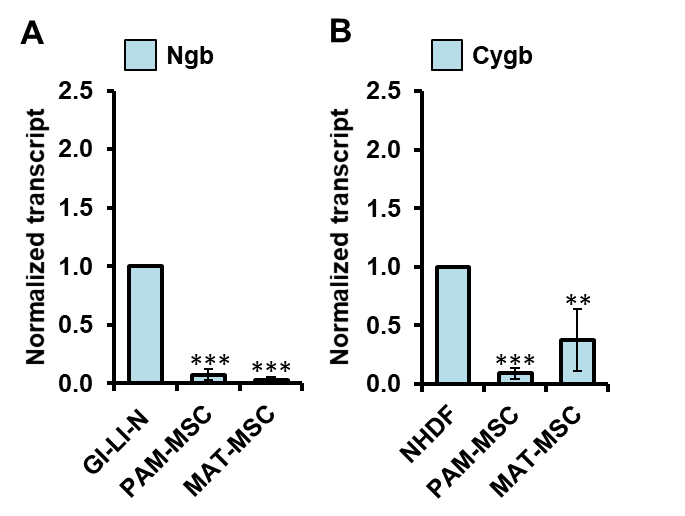


**Figure S2.** Quantitative RT-PCR analysis of the expression of neuroglobin (Ngb) (**A**) and cytoglobin (Cygb) (**B**) genes normalized to those of the neuroblastoma cell line (GI-LI-N) and normal human dermal fibroblasts (NHDF), respectively; means ± SEM of 3 independent measurements (biological replicates) each carried out in 3 technical replicates; **, P < 0.01 and ***, P < 0.001.


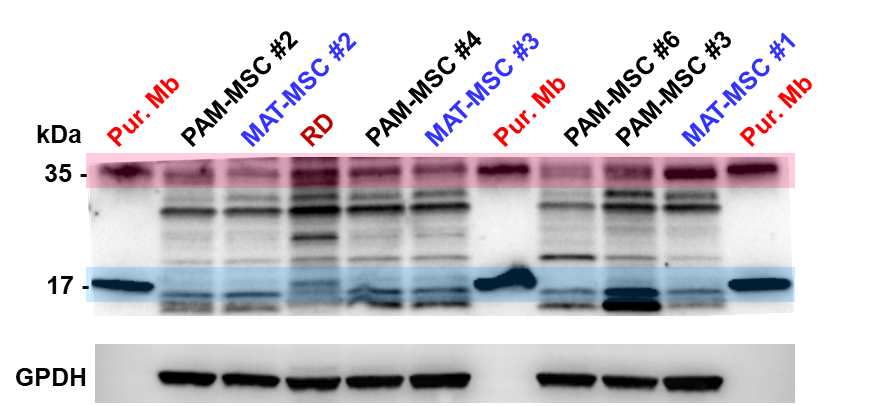


**Figure S3.** Uncropped Figure 1 D shown in the main text. Representative western blotting of different preparations of amniotic placental membrane (PAM-MSCs) and mammary adipose tissue (MAT-MSCs); RD, human rhabdomyosarcoma-derived cell line; Pur. Mb, purified horse heart myoglobin. Several commercially available anti-Mb were used to limit cross-reactions but unsuccessfully. The protein bands highlighted with the light red and blue strips are the *bona fide* dimeric and monomeric Mb respectively as confirmed by mass spectrometry (see Figure 2 of the main text).

**
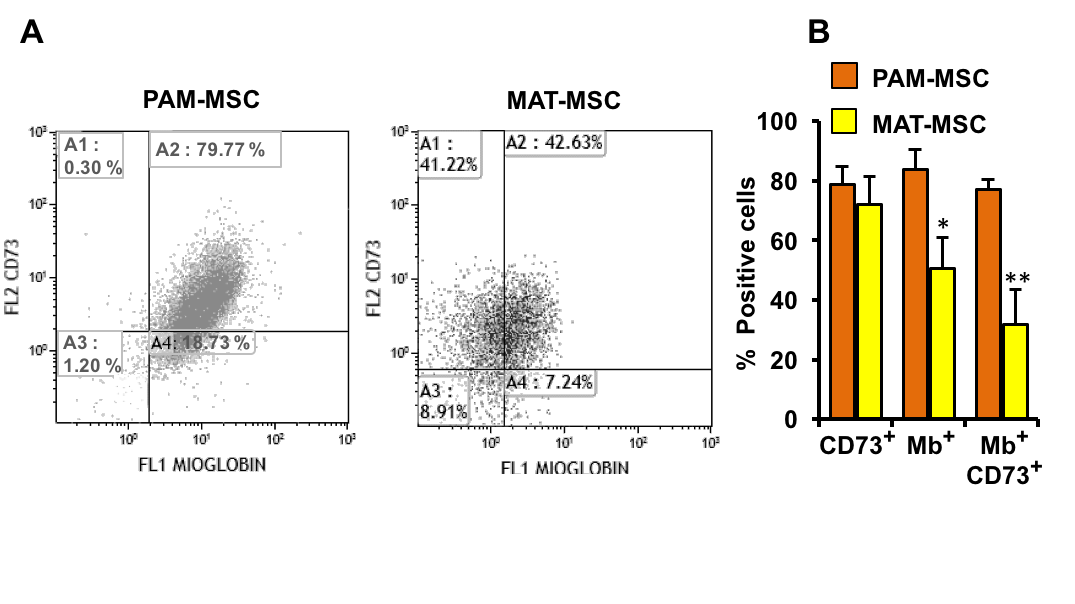
**

**Figure S4**. Co-expression of Mb and CD73 in PAM- and MAT-MSC. (A) Comparative flow cytometry dot plots; MSCs were stained with PE-conjugated anti-CD73 and FITC-conjugated secondary Ab for anti-Mb (see Materials and Methods for details). (B) Statistical analysis of the percentage of positivity in PAM-MSC and MAT-MSC to Mb and CD73 alone or combined; the values are means ± SEM of three biological replicates; *, P < 0.05 and **, P < 0.01.

**
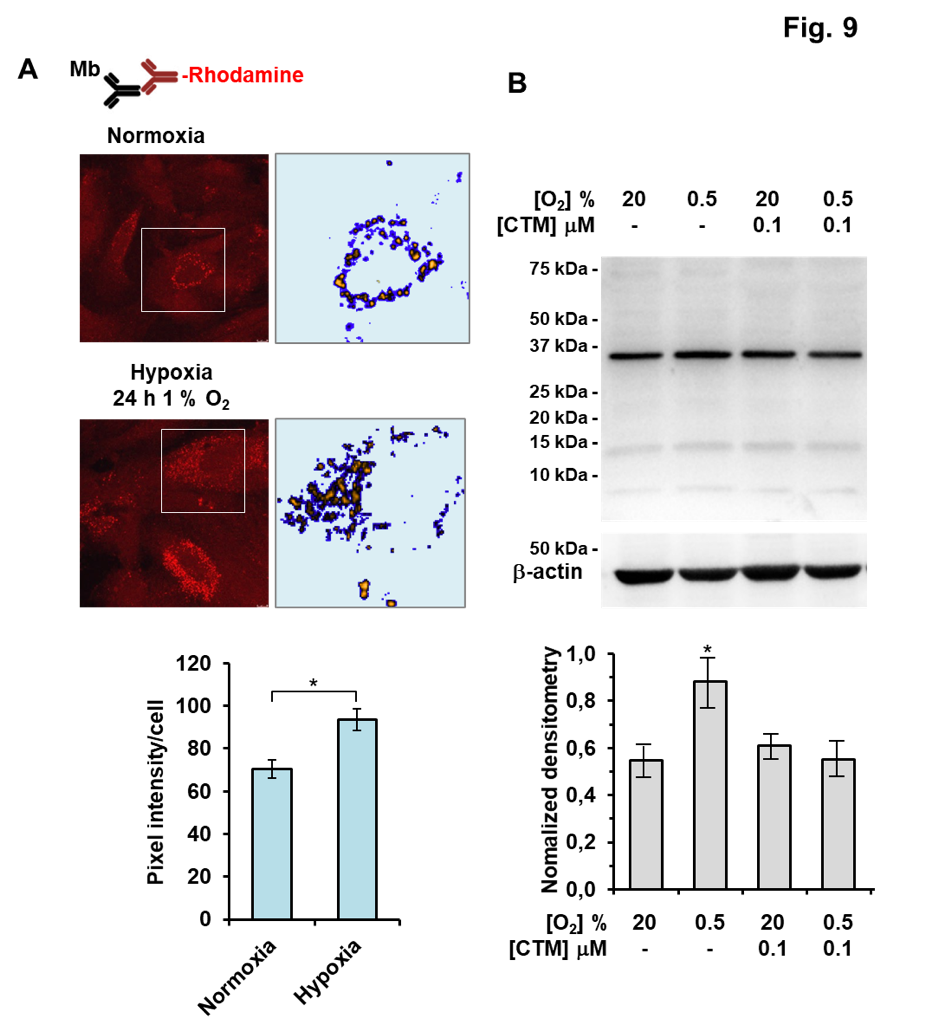
**

**Figure S5. Effect of hypoxia on the Mb expression in PAM-MSCs.** Cells were incubated at 0.5-1.0 % O_2_ for 24 h. (A) Immuno-cytochemistry treatment and confocal microscopy analysis for detection of Mb (see Materils and Methods for details). The upper panels show representative images of immune-detected Mb under normoxic and hypoxic conditioning; digital magnification of details are shown aside after false colour rendering and identical threshold to remove background. The histogram at the bottom show the intensity per cell of the Mb-related fluorescent signal; the values are means ± S.E.M. of three independent preparations of PAM-MSCs; at least 10 different optical fields were unbiasedly selected each containing 8-10 cells for every single assay; *, P < 0.05. (B) Representative Western blot for Mb detection of total protein lysates of PAM-MSCs incubated for 24 h at 20 % or 0.5 % O_2_; where indicated chetomin (CTM) was present during the incubation. The histogram shows the normalized densitometry of the 35 kDa band; the values are means ± S.E.M. of three independent preparations of PAM-MSCs; *, P < 0.05.
